# Human Enough: A Qualitative Study of Client Experience With Internet-Based Access to Pre-exposure Prophylaxis

**DOI:** 10.2196/22650

**Published:** 2021-07-05

**Authors:** Shana D Hughes, Kimberly A Koester, Edvard Engesaeth, Merissa V Hawkins, Robert M Grant

**Affiliations:** 1 Department of Medicine University of California, San Francisco San Francisco, CA United States; 2 Nurx San Francisco, CA United States

**Keywords:** telehealth, internet, HIV, pre-exposure prophylaxis (PrEP), client/user experience, qualitative

## Abstract

**Background:**

HIV pre-exposure prophylaxis (PrEP) is a way to prevent HIV infection using antiretroviral medications. However, common barriers to PrEP engagement include lack of access to prescribers; discomfort seeking sexual health services; and racism, homophobia, and transphobia in medical contexts. Key populations (eg, communities of color, young men who have sex with men, and transgender women) are underrepresented in terms of PrEP uptake in the United States. Nurx is an innovative company that has offered internet-based access to PrEP since 2016.

**Objective:**

In this study, in partnership with Nurx, we aim to explore clients’ experiences of digital PrEP access—including the difference made by the telehealth format—and to understand whether Nurx helped reduce barriers to PrEP.

**Methods:**

Electronic chart review and semistructured interviews were conducted with 31 PrEP requesters from California, Florida, Illinois, and New York. Interviews were recorded, transcribed, and subjected to inductive and deductive thematic analysis.

**Results:**

Some interviewees reported initial skepticism about whether a web-based PrEP service could be legitimate or feasible. Despite this, most clients were effusive about their eventual Nurx experience, and many reported that Nurx eased barriers to PrEP access through the availability of knowledgeable, willing prescribers and minimizing embarrassment and discrimination. Our analysis suggests Nurx produced satisfaction by achieving an acceptable balance between 2 client desires: *efficiency* and *humanity*. Efficiency encompasses the simplicity, speed, and convenience of obtaining PrEP, both regarding the Nurx process itself and in comparison with in-person encounters. Humanity covers clients’ wish for personalized, responsive interaction and a feeling of connection or care. Nurx’s messaging platform was crucial to manifesting these qualities and was largely interpreted through the familiar frame of texting. Clients conceived efficiency and humanity as inversely related in a commercial enterprise and varied in the particular balance they felt was optimal. Those who wished for slightly more humanity than the service afforded used the concept of a *trade-off* to explain why Nurx remained appealing.

**Conclusions:**

Our findings augment evidence that internet-based PrEP provision can broaden access to this HIV prevention strategy. This important finding, notwithstanding a few provisos, merits mention. Telehealth, as practiced by Nurx, was still dependent on culturally competent medical providers as system *inputs*, and the very technology used to overcome access barriers (ie, the internet) generated new hurdles for some clients. Furthermore, clients did not interpret Nurx in a vacuum: their past experiences and the social and structural context mattered. Finally, only granular inquiry revealed precisely *how* Nurx satisfied clients whose experiences and preferences fell within a particular range. Extrapolating from this, we urge scholars not to fetishize technological solutions but rather to interrogate the ways in which any intervention’s design works for certain kinds of patients.

## Introduction

HIV pre-exposure prophylaxis (PrEP) is a way to prevent HIV infection using antiretroviral medications. The US Food and Drug Administration has approved 2 pharmaceutical products for use as PrEP: tenofovir disoproxil fumarate combined with emtricitabine (for all populations) and tenofovir alafenamide combined with emtricitabine (for adult men and transgender women). PrEP is taken by individuals who are HIV-negative and has been proven to be clinically effective among multiple target populations [1]. Widespread use has already been associated with a significant reduction in new infections in certain local epidemics [2,3]. However, to realize the promise of this biobehavioral HIV prevention strategy, those who are most at risk must opt to take the drugs, and it is well known that some key populations (eg, communities of color, young men who have sex with men, and transgender women) are underrepresented in terms of PrEP uptake in the United States [4,5]. Documented barriers, which may be particularly acute among such populations, include lack of awareness of PrEP, lack of access to or difficulty accessing care, fewer resources to pay for out-of-pocket expenses associated with PrEP (eg, laboratory work), same-sex stigma and racism in medical contexts, and immigration status [6-8].

Telehealth is defined by the US Health Resources and Service Administration as “the use of electronic information and telecommunications technologies to support and promote long-distance clinical health care, patient and professional health-related education, public health and health administration” [9]. This modality of care is not new [10] but is drawing attention as one way to potentially circumvent barriers to PrEP access, especially in the era of COVID-19 when access to specialty care services and care in general is compromised [11]. A recent review [12] noted that PrEP telehealth interventions are being deployed to various ends. Some efforts seek to enhance access among patients who are geographically isolated or encounter challenges locating a knowledgeable provider; others facilitate clinical consultation and/or aim to build capacity among medical providers who may be willing to prescribe PrEP but desire support [5,13-16]. These endeavors are largely the product of partnerships between government, academic, and community stakeholders, and early results suggest that telehealth-based PrEP is feasible and acceptable and may reduce some barriers to access [12], although detailed data on the experiences of patients are scarce.

Private companies have also entered the PrEP telehealth market. One such company in San Francisco, California, called Nurx, offers web- and app-based counseling, prescription, and home-delivery services for PrEP. Nurx began to offer internet-based access to birth control in 2015, expanded to include PrEP a year later, and is now operating in 29 states and the District of Columbia. Qualitative researchers at the University of California, San Francisco (UCSF) partnered with Nurx to explore the possibilities raised by untethering PrEP from traditional brick-and-mortar medical contexts. The initial research objective is to understand how clients make sense of their experience, with special interest in what *difference*, if any, the web-based format makes.

## Methods

This research employed a mixed methods design, conducting an electronic chart review and semistructured telephone interviews with clients who requested (although did not necessarily receive) PrEP through Nurx. Sampling so-called *PrEP requesters* was intended to enable the examination of both the experience of completing the envisioned service cycle and the barriers or circumstances that might prompt a prospective Nurx user to initiate but desist from engagement with the service.

Nurx personnel used the platform’s asynchronous messaging system to contact all users who had requested PrEP in California. This outreach provided a brief, initial description of study procedures and purpose and requested that users interested in learning more respond with *Yes*. A user’s response authorized UCSF interviewers to directly correspond with potential participants through the messaging system and via email (users needed an email address to create a Nurx profile, so this did not limit participation). Participants received a study information sheet via email and asked questions about the research before arranging a telephone interview. Interviewers familiarized themselves with consenting interviewees’ cases through chart review. Although we do not directly report data gained through this step in this paper, it was nonetheless important, as it sensitized us to issues that might surface during the interview, such as service gaps, challenges with billing, insurance, or delivery. In some cases, chart review—which included messages exchanged between the clients and the Nurx team—provided a way to triangulate interviewees’ accounts. This general procedure was repeated for PrEP requesters in Florida, New York, and Illinois.

The interview guide covered previous knowledge and/or use of PrEP; learning about Nurx; interacting with the web-based platform and Nurx personnel; and receiving, taking, and desisting from PrEP, as relevant. In addition, the guide explored interviewees’ motivations for requesting PrEP and sexual practices. Interviews were conducted by KAK and SDH lasted for not more than 90 minutes and were digitally recorded with permission. The interviewees received a US $50 electronic gift card. Data were gathered from April to August 2017, with interviewers jointly debriefing as interviews were conducted. The Institutional Review Board at the UCSF approved all the research procedures.

Interviews were transcribed verbatim, and transcripts were uploaded to MAXQDA 12 Plus [17], a qualitative data analysis software package, to facilitate analysis. Both interviewers participated in the coding process, with transcripts divided evenly between them for first-pass thematic coding. Both deductive and inductive approaches were used to identify salient themes [18]. Deductive codes were drawn from the interview guide (eg, *learned of Nurx* and *adherence*) and the literature (eg, *insurance* and *stigma*). Inductive codes emerged from a close reading of the text itself (eg, *approachable* and *automated messaging*). After the first coding pass, interviewers jointly refined code definitions, and each interviewer reviewed the coding done by her teammate, having the option to confirm the code applications or modify them in accordance with the revised codebook. Instances in which the revised codebook did not resolve coding discrepancies were examined, and consensus was achieved through discussion. This paper draws on thematic analysis of materials from 31 PrEP requesters in California, New York, Illinois, and Florida. Exemplary quotes in the sections that follow are attributed to interviewees by number to protect their confidentiality.

## Results

### Sample and Service Flow

Interviewees were predominantly male, aged 30 years or younger, racially and ethnically diverse, highly educated, and identified as gay. Nearly all had insurance, whether public, private, or through parents (Table 1).

**Table 1:** Participant demographics (N=31).

Characteristic			Participant, n (%)
**Sex**			
	Male	26 (84)	
	Female	5 (16)	
**Age (years)**			
	18-30	25 (81)	
	31-65	6 (19)	
**Race or ethnicity**			
	White (non-Hispanic)	10 (32)	
	African American (non-Hispanic)	4 (13)	
	Hispanic or Latino	8 (26)	
	Asian	3 (10)	
	Mixed or more multiracial	5 (19)	
**Insurance**			
	Uninsured	1 (3)	
	Medicaid	8 (26)	
	Parents (unspecified)	5 (19)	
	Employer based	11 (35)	
	Kaiser	4 (13)	
	ACA^a^ Plan	2 (6)	
**Education**			
	High school or less	3 (10)	
	Some college, vocational training	9 (29)	
	Undergraduate degree or more	19 (61)	
**PrEP** ^b^ **status**			
	Never taken	12^c^ (39)	
	Currently taking	17^d^ (55)	
	Took and stopped	2 (6)	
**Duration on PrEP (for those who had ever taken PrEP)**			
	Less than 6 months	9 (47)	
	More than 6 months but less than 3 years	7 (37)	
	3 years or more	3 (16)	

^a^ACA: Affordable Care Act.

^b^PrEP: pre-exposure prophylaxis.

^c^Includes 2 who were awaiting shipments or laboratory orders from Nurx at the time of interview.

^d^Even if no longer receiving pre-exposure prophylaxis from Nurx.

**Figure 1:**
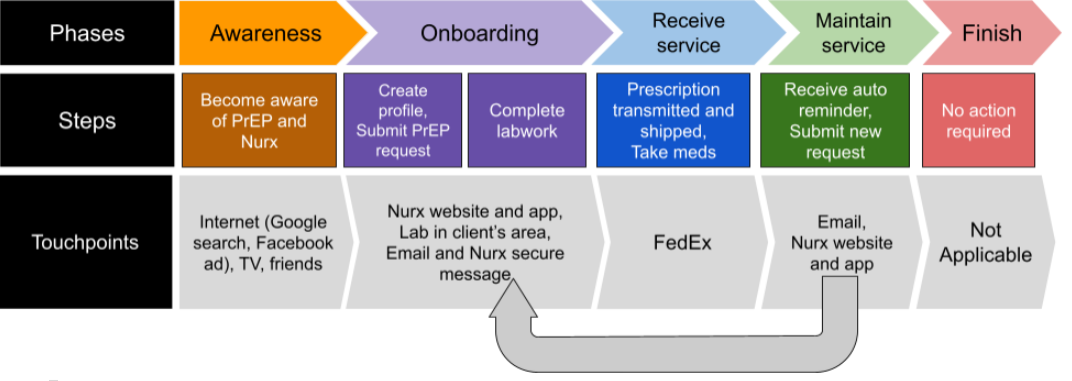
Simplified Nurx client journey map. PrEP: pre-exposure prophylaxis. TV: television.

Owing to the novelty of web-based access to PrEP, it is helpful to sketch the Nurx service flow before engaging with the thematic analysis. To do this, we drew from interviewees’ descriptions (supplemented with details provided by Nurx staff) to create a simplified journey map of the Nurx client experience, as it was at the time of data collection (Figure 1) [19]. In service design, a client journey is seen to have general phases: awareness, onboarding, receiving the service, maintaining the service, and finally, if necessary, finishing [20].

In the case of Nurx, clients had to become aware of both PrEP and Nurx. Onboarding included multiple steps. Users created a profile and then initiated a *PrEP request* by answering a web-based questionnaire about sexual behaviors and other HIV-related risks. Those with health insurance also typically provided this information by using a mobile phone to take a photo of their insurance card and uploading that photo to the Nurx site. Personnel at Nurx reviewed this information, and a clinician then sometimes initiated a discussion with the client about the motivation for the PrEP request before sending electronic orders for HIV and other tests (including renal function, hepatitis B and C, sexually transmitted infection [STI], and pregnancy screening, per Centers for Disease Control and Prevention guidance) to a laboratory in the client’s area. Laboratories relayed results back to Nurx electronically, and these were reviewed by a Nurx physician.

For clients with nonreactive HIV tests, the first step in the *receiving the service* phase was the transmission of a prescription for PrEP to a pharmacy. However, this happened on *the back end*, meaning it was invisible to clients. They received a message from the physician through the Nurx site that included the results of their laboratory tests, notification that they were being prescribed Truvada, dosing guidance, and other recommendations (eg, PrEP does not protect against STIs other than HIV and so condom use is still recommended). The 3-month prescription was usually shipped directly from the pharmacy to a client-provided address via FedEx. At the time of data collection, the clients had to sign for the package. Once the medication was delivered, the client could begin taking PrEP.

Maintaining the service started with an automated message from Nurx, sent to clients several weeks ahead of prescription renewal time, informing them of the need to submit a new PrEP request through the site. The new PrEP request then generated laboratory orders, and the cycle started again. Clients who did not submit new requests or failed to complete their laboratory tests typically received additional reminders. Prescriptions would be renewed if and when clients’ renal function was within the normal range and the HIV test result was negative.

Only 4 participants in this study had experience with finishing. Of 4 participants, 2 men had to stop using the service because of changes in insurance coverage. Another participant stopped using PrEP entirely. The fourth participant might more appropriately be described as *lapsed*, as he had been unable to complete the lab work necessary for his prescription refill. Irrespective of the motivation, finishing did not require the client to do anything; they could simply stop responding to Nurx messages. Even so, several participants used the messaging system to let Nurx know that they would no longer be using the service.

### Client Experience: Satisfaction After Overcoming Skepticism

Most interviewees reported being extremely satisfied with Nurx services and peppered descriptions of their experiences with adjectives such as *friendly*, *professional*, and—above all—*convenient*. Illustrative of the general trend, P04 summarized:

For someone whose life is busy and unpredictable, this service was like a dream. This really was perfect for my needs.

Such expressions of satisfaction were common, even among the 10 interviewees who, for various reasons, had not actually accessed PrEP through Nurx or were no longer using the service. As one participant who had moved to a health maintenance organization and found Nurx was no longer covered said:

Long story short—any company that is trying to cut [insurance- and cost-related] barriers down, I really root for. So, the only sad thing [about not being able to use Nurx] is that they’re going to lose an active user, now. I want their numbers to be up.

P25

The pervasive satisfaction was striking because interviewees often related having to overcome an initial hurdle as they contemplated using Nurx: wondering if the service could possibly be *legit* or whether it was *too good to be true*. This was partially driven by general skepticism about internet content and web-based interactions and was one way in which the web-based format exerted a unique influence on clients’ experiences. As such, the telehealth option presents a clear contrast to brick-and-mortar clinical interactions. One interviewee explained:

Getting a prescription online, it doesn’t seem super legitimate at first...I just had to make sure that...people were using [the website], and...it was working for them before I put my personal information on there...Because I don’t want to just hand that to some random person on the Internet.

P28

In general, interviewees recounted engaging in *research* intended to ascertain the service’s legitimacy. This could include evaluating the Nurx site itself, accessing user reviews, conducting web searches, and reading about the service on trusted news and information sites. In a typical example, one interviewee listed multiple sources that bolstered his confidence in Nurx:

[*The company] had articles talking about it. [The website] looked clean You know, people posted their experiences about it. So, I knew...it’s not some scam website that just wanted my health information or something like that*. [P21]

In addition to general concerns about the veracity of web content and the legitimacy of the service, the idea of web-based PrEP access gave some interviewees pause because of what they perceived as potential shortcomings of telehealth. For example, one participant was hesitant because:

[Nurx] could very easily not get the whole clinical idea of what’s going on with the patient because you’re not actually laying eyes on them. And, you know, to me, that just seemed, like, really weird, because I [hadn’t] really explored telehealth or anything like that at that point. So, it was just really weird to me. But I was just, like, okay, whatever. I can’t get it through my doctor and I really want to get it, so let’s reach out through this and try it.

P24

PrEP-specific logistical questions also surfaced. Some interviewees who had taken PrEP before learning of Nurx voiced a perception that it might be more complex than telehealth could accommodate. As one participant said:

I was like, “This is such a cool idea.” There was, I think, not skepticism, more like disbelief because it was so unlike anything that I’d seen before and I couldn’t really conceptualize, “Well, how does this really work?” Because this is something that seemed too complicated.

P04

Another interviewee, who had been taking PrEP for 2 years before discovering Nurx, said his initial concern had been that:

you have to have lab work. So I didn’t understand how that would work out [online], until I actually went to their website and read the information.

P36

It should be noted that the doubts reported here arise *because of the web-based format*, that is, the very feature that made the service novel and attractive (web-based access and its convenience) also raised barriers to its use. Clients who experienced this (not all did) had to seek out specific information and allay particular suspicions before they felt that PrEP access through telehealth was a truly viable option. These users became sufficiently convinced that Nurx was *legit* (and the system could work) to register and submit requests.

The foregoing notwithstanding, Nurx’s service was not flawless. Especially when pressed (eg, “Is there anything at all you would change about the service?”), clients mentioned issues in various parts of the service flow. Some participants had trouble finding the area on Nurx’s website that dealt with the PrEP service (P01, P17, P19, and P28), and others explicitly wished for an app (P01, P07, and P21—there might have been more mentions of this had Nurx not rolled out an app during data collection). Some clients had problems at the laboratory they visited (P14, P16, P17, and P25), whereas others encountered challenges with insurance (P02, P15, P20, and P21). Shipping or receiving medication was a frequent source of trouble, especially the FedEx signature requirement (P10, P13, P23, P25, and P36; P02 and P15 also had problems with lost shipments).

To provide more context on these issues, we highlight the case of P07. After signing up with Nurx, he had made a laboratory appointment for the following week and then encountered what he characterized as *a little bit of a rough patch*. This seemed to be a mild description of what was actually one of the most trouble-ridden service journeys in the data set. At his laboratory appointment, a data entry error meant that the staff could not locate the appointment. He made a second appointment but forgot his confirmation number; the laboratory staff said they were unable to help him without it. He made a third appointment a few days later, and that appointment was short and smooth. After subsequently receiving confirmation from Nurx that they could prescribe PrEP for him, he had problems setting up an account with the pharmacy handling the dispensation and delivery. There were issues with his insurance and confusion about how much he would have to pay for the medication. However, at most of these junctures, he mentioned messaging Nurx through the platform and receiving help. He described the challenges he had confronted as “purely, like, circumstantial. It wasn’t the process by any means.” When asked how the experience of using the service compared with his expectations, he said:

It’s exceeded actually. I think it’s made getting this done...extremely stress-free other than the little hiccup that I had the first time.

P07

This is illustrative of one of the strongest trends in the data set: although interviewees could recount challenges with the service, their overall assessment of Nurx was positive. Many participants frequently recommended it to friends and sex partners, and several voiced solidarity with the company. For example, P21 said:

I love like the whole startup thing, you know...I want to see Nurx sort of succeed.

Indeed, only one interviewee’s assessment (P24) could reasonably be described as lukewarm overall. Partly because of this, in the rest of this paper, we will pivot to interrogate the pervasive satisfaction that colored the narratives, understanding *how Nurx services produced this* and *for what kinds of clients,* as this can provide a window onto which barriers telehealth actually alleviates.

### Client Experience: The Production of Satisfaction

At base, we consider Nurx to have produced satisfaction by achieving an acceptable balance between 2 fundamental client desires, which we have termed *efficiency* and *humanity*. *Efficiency* encompasses the ease of obtaining PrEP, both in terms of how simple and convenient the Nurx process itself is, and the ability to avoid disliked aspects of other ways of getting PrEP. *Humanity* covers the clients’ wish for personalized, responsive interaction and a feeling of connection or care. We draw from interviewees’ narratives to illustrate these dynamics and their interplay as well as highlight the influence of the web-based format.

Efficiency was the way interviewees most clearly contrasted Nurx with traditional clinical contexts. The greatest gains in efficiency accrued when users who otherwise encountered obstacles locating a knowledgeable, willing, available prescriber were able to access PrEP through Nurx [21]. One interviewee, for example, described PrEP as barely a thing where he lived, adding that few health care professionals seemed aware of it. Several of this interviewee’s friends had related that:

their doctor doesn’t know anything about it, and they’re not comfortable prescribing it to them. So, they usually get referred out to the [AIDS Health Foundation] in town...So, I was actually going to go there to try to get a prescription, but they were booked out, like, six months or something for appointments.

P30

Shortly after learning of the 6-month wait, this interviewee discovered Nurx had expanded its services to his state and he signed up *right away*.

Even users who did not face such extended wait times for appointments described the ability to start the PrEP process at their convenience (ie, by providing initial personal and insurance information through Nurx’s web-based portal) as removing a significant constraint. In addition, the quasi-anonymity of submitting answers to behavioral screening questions on the web was highly valued. One participant noted that going to the doctor “for, like, sexual type, gynecologic visits” typically provoked nervousness and stated:

I feel that weird shame

P06

Another said:

If you were speaking to somebody face-to-face about [the] same list of questions that a medical practitioner will ask you [about PrEP], say,...“Do you have unprotected sex with people that may or may not be drug users?” Face-to-face with someone you are not familiar with, that’s not a very comfortable question to answer. Now if you get those questions from an online interface, it’s a lot easier because there’s a potential of not being judged. You’re talking to a person, but you’re talking to a person via email or vis-à-vis the messaging system. It makes you much more comfortable and much more honest.

P03

Nurx’s method of obtaining information on patient sexual practices reduced embarrassment, awkwardness, or shame, which these interviewees recounted fearing or having experienced with previous providers. We categorized this as *efficient* because it avoids unnecessary and unpleasant elements of obtaining PrEP in person.

The service flow often went smoothly. When it did not, however, assistance from Nurx—human intervention—was welcomed. For instance, many clients reported great concern over issues of cost and billing and a desire for personalized, real-time responses to questions about these issues. One interviewee reported that his only concern about obtaining PrEP through Nurx had been uncertainty around the cost. After receiving an initial bill of several thousand dollars for a 3-month supply of PrEP, in *sticker shock*, he contacted Nurx and discovered there was a problem:

with how my insurance was filing it or something. So, I did have to go back and forth with them a little bit to figure that out. And the price did go down at some point, but it was still over—I think it was, like, $1500 or something was the actual amount that I had to use [the Gilead Patient Assistance Program card] to pay.

P30

In such cases, Nurx’s ability to respond quickly and individually was key to clients’ satisfaction with the service (it bears noting that in this instance, Nurx had proactively signed this user up for the copay card to begin with). With very few exceptions, these interactions occurred through the platform’s messaging system. Indeed, when clients talked about the *service* Nurx provided, they mostly discussed messaging. Hence, we examined clients’ perceptions of messages in detail.

### Messaging

Several interviewees spoke of previously engaging in messaging with a provider through electronic portals that were a part of larger health systems, so this was not always a novel experience. One interviewee reported using such a system to email his primary care provider (PCP), asking to “get [PrEP] started.” Once the interviewee learned that he would need to see his PCP in person before starting PrEP, he “decided to try and go through Nurx because [the health system’s] availability for check-ups and just anything like that is really impacted” (P08). Although messaging is not necessarily unique to telehealth, what a patient can expect to accomplish by messaging Nurx versus messaging a PCP is likely different.

As previously mentioned, some interviewees noted the potential for feeling less judged through a web-based interface. When asked why this was so, one user (P03, quoted earlier) reflected on providing answers to the sexual behavior questions:

Well, people say that emotions or intention is a little bit [harder] to read through text message than it is face-to-face. I honestly feel it kind of applies to what’s going on.

P03

Interestingly, however, messaging was described as being able to both block the communication of emotion (as mentioned earlier) *and* convey the sense that Nurx was friendly and approachable. As one client explained:

The way they write their messages is very cheerful...They addressed you by name like, “Hi, G. my name is so and so and I’m here to collect your insurance information.”...I guess I expected it was going to be much more robotic, like, “Please submit such and such information by this date.” It wasn’t like that at all. It was much more personable, like you were just texting a friend back and forth or something.

P13

In this sense, the messaging platform figures in clients’ narratives as a tool that Nurx can deploy in the service of both efficiency (*simple and convenient*) and humanity (*just texting a friend*).

Many interviewees described the message system as similar to texting and, thus, familiar. One participant explained why this works in Nurx’s favor:

The thing that I know is that millennials hate to be inconvenienced. If...they don’t have to actually go and talk to someone, I think they will be more inclined to see the process through. Because [also,] the process mimics that of a texting platform that they’re used to being on. It mimics that of a social media platform with the way you can post photos to your physician.

P02

However, some interviewees directly addressed the use of automated messages, seeing them as distinct from those that felt like *texting a friend*:

I can definitely, in the correspondences, tell if it’s a form or if it’s casual. Obviously, it helps if you ask, like, a bespoke question and receive a bespoke answer. Clearly, that’s not coming from an AI bot. But as far as automated, like, “Hey. Thanks so much for filling out your application. We’re running at full capacity right now and hope to have your results in the next day or so.”...No one sat behind a computer and just casually wrote that before going to the bathroom. You know? That’s read over many times by a lot of people, and they thought this was the best they could say.

P23

And how do you feel about that, that there are automated responses going out?

Interviewer

It’s a service. It makes sense that it would be automated.

P23

Although this interviewee had received both *form* and *casual* responses from Nurx, here, he clearly constructs the feasibility of the service in its web-based format as dependent upon some degree of automation, that is, to remain in business, Nurx as a commercial entity had to serve a sufficient number of clients, which automation made possible. Another interviewee echoed this notion, suggesting that clients sometimes had to accept interactions that felt somewhat *disconnected*. For example, when *specific questions* arose (eg, around insurance or laboratories), messages could be answered by various customer service representatives working the message queue, making him feel:

In the flow, it’s kind of like, I don’t have a specific person to talk to...But, it’s an interesting sort of trade-off. Because, at the same time, it was so easy to go through that and...I understand that as being the tradeoff. For like, the ease and simplicity, vs. like, having that like, specific person to contact and like, them being like, readily available.

P25

This client’s construction of a *trade-off* crystallizes a more implicit notion that was common among client narratives: that efficiency and humanity are inversely related, such that as efficiency increases, humanity decreases and vice versa. The challenge for Nurx was to provide the efficiency clients sought while remaining human *enough*.

A case in point is the experience of actually receiving the medication. Clients described home delivery as an attractive feature of the service, imagined as consummately efficient. However, it was also a touchpoint at which efficiency could falter in practice because of the FedEx signature requirement in effect during data collection (it has since been removed). That a human, physical presence was necessary to complete a transaction touted as virtual was both a conceptual and practical obstacle for many users and detracted somewhat from the seamless way Nurx aimed to fit into its clients’ busy, highly mobile lifestyles. Some users had to identify alternative delivery addresses; others consistently had to travel to FedEx locations (or in one case, a local pharmacy itself) to collect their prescriptions. It might be surprising that clients accommodated such demands, given the premium they placed on convenience and efficiency. Our analysis suggests that the concept of a trade-off may help explain. Specifically, it seems that the clients we interviewed were willing to take such actions in exchange for the convenience offered by the other phases of service flow and as long as emergent obstacles throughout the client journey were handled with sufficient humanity. Indeed, it was not only clients who had no other way to obtain PrEP that used the service; those who might otherwise have obtained PrEP from PCPs often chose to remain with Nurx as well. As summarized by one client:

Despite all of these complications...getting access to this drug and staying on it—because it has been kind of difficult for me—...each person I’ve talked to [at Nurx] has, to me, seemed very genuinely concerned and sympathetic and really willing to help me out.

P15

This makes plain the importance of the balance that Nurx strikes between efficiency and humanity.

### Efficiency-Humanity Balance

The foregoing notwithstanding, within the data set, clients expressed varying levels of tolerance and appreciation for efficiency and humanity. Some gave the impression that they were close to needing more of the latter than Nurx provided. One interviewee (P01), an ardent proponent of self-monitoring and quantification, had negotiated to have the Nurx physician submit standing orders for STI screening at the laboratory where he did the required testing. This meant that he did not have to wait the customary 3 months; it is unclear how failing to obtain this from Nurx might have impacted his satisfaction with or continued use of the service. Another participant explained that:

the problems that I have with [the service] aren’t really, like, major issues

but he recommended Nurx “be more personalized” in their communication with clients, because:

using my name isn’t going to really cut it. And very rarely do we get into, like, personal conversations unless I initiate something...yes, the anonymity helps, because you just have a little picture in a box [when chatting]. That’s great. [But] that could not actually ever be a person. You could be in China, like, cutting or pasting on 27 different things like everybody expects is happening through telehealth.

P24

Thus, a lack of *personalization* led the participant to question whether there is *a person* on the other end of the chat at all or whether the person really is who they claim to be. Indeed, never physically seeing the physician seemed to have left a kind of residual uncertainty for several users. One asked:

Is [Dr. X, a Nurx physician] a real person?...There’s, like, a picture [on the site] of a really pretty lady, and it seems like she might be a stock image or something, you know?

P30

Yeah [...] she’s a real person.

Interviewer

Okay. I’ve been wondering that. Okay. [...]

P30

Was there something else, about the way that she was interacting with you, that made you wonder about that?

Interviewer

No. It was always, like, a really personalized response. So, I knew it wasn’t coming from a robot. Yeah. I just kind of—I think it was the photo.

P30

This skepticism about the true identity of Nurx service providers echoes the initial doubts some users expressed about the service itself, in that it is engendered by the web-based context of the interaction. However, as with these initial doubts, the discomfort this produced was not sufficient to dissuade these interviewees from using the service.

In contrast to interviewees who seemed to have their needs for *humanity* barely met by Nurx, others appeared to have a much greater tolerance for *efficiency* than the service required, especially if they had existing relationships with other care providers. For these users, a sense of emotional resonance in Nurx’s messages or personal connection with the PrEP provider was not that important. For example, in contrasting web-based and face-to-face clinical encounters, one participant said:

[Online is] not as personable, you know, but that’s honestly not a big deal to me...I like to get in there, get everything taken care of, and go, you know? We don’t have to, you know, ham it up and just have this great, friendly relationship.

P27

These data suggest that users arrive at the Nurx clinical encounter with a set of individual preferences and needs regarding the patient-provider relationship, an orientation that circumscribes a range within which interactions will be considered acceptable. At one end, some clients seemed to wish for slightly more humanity than was built into the service’s default operation; at the other end, clients were satisfied though basically uninterested in having a relationship with Nurx that went beyond the transactional. Similarly, Nurx is configured in ways that enable a range of service-user interactions (those encompassed within the particular efficiency-humanity balance struck by the company) and make others less likely or impossible. When the user and company ranges overlap, clients are likely to judge the resulting interactions as satisfactory, as long as their end goal (eg, obtaining PrEP) is also achieved. On the basis of our data, the ideal Nurx client is a person who has access to and is relatively comfortable using the internet, did not experience or was able to overcome initial skepticism, feels their schedule is busy enough that all kinds of real-time appointments are a chore, may wish to avoid face-to-face discussions of sexual practices, and/or does not require an intensely personalized relationship with the PrEP provider.

## Discussion

### Nurx: Telehealth Considerations and Lessons

Although telehealth-based PrEP provision is increasingly mentioned as a potential way to broaden access to this HIV prevention strategy [22] and a handful of innovative interventions have appeared in the literature [12], fine-grained data on the patient or user experience are scarce. The partnership between UCSF researchers and Nurx enabled the collection of qualitative data to shed light on this topic from within a commercial environment. Our analysis led us to explain client satisfaction as resulting from the balance the company struck between what we have termed *efficiency* (convenience, ease, and automation) and *humanity* (personalized interaction and care). The concept of a *trade-off* explains how certain highly valued parts of the service (eg, convenient, flexible service initiation; quasi-anonymous discussion of potentially sensitive sexual health topics; and messaging platform and practices) helped users accept other relative inconveniences (eg, delivery issues or, in some cases, presenting for follow-up laboratory testing [23]). In addressing the important questions of whether Nurx provided a way to overcome common barriers to PrEP access and what difference the web-based format made, we ground the discussion firmly in our data while answering with an eye toward telehealth more generally. In light of the massive surge in interest and use of web-based formats for medical appointments prompted by the current global COVID-19 pandemic, we hope that scholars and practitioners will find the insights and questions we raise transferable [24] and valuable.

We found evidence that Nurx allowed patients to locate a knowledgeable and willing prescriber (as long as they lived in a state served by the company). In addition, receiving PrEP services from Nurx enabled users to avoid stigmatizing or embarrassing encounters with providers. These outcomes are important and should not be minimized, particularly as they manifested among a diverse patient population that included young men of color who have sex with men, a group disproportionately impacted by HIV [25]. That impact notwithstanding, in assessing Nurx as an intervention, we should distinguish between outcomes that are produced by, as opposed to merely facilitated by, telehealth. Connecting providers and patients despite the geographic distance between them and reducing the burden of medical appointments (by removing the need to travel to and from) are inherent capabilities of telehealth. In contrast, that Nurx personnel and physicians were experienced as nonjudgmental during potentially sensitive *conversations* is not *produced by* the web-based format, in the sense of being guaranteed simply by virtue of happening on the web. While technology enabled users to interact with Nurx personnel without being face-to-face (which was said to remove some potential for embarrassment), the content and tenor of those exchanges also certainly influenced users’ comfort level. Both Nurx’s underlying company philosophy and their experience of providing oral contraception may have contributed to their capacity to create interactions users described as nonjudgmental and sex positive. If the providers on the other end of the messaging platform had lacked cultural competence in providing sexual health services [26], it is unlikely that users would have felt so at ease. This points to the importance of particular types of providers and messaging as components of a telehealth system [21].

As mentioned earlier, although Nurx helped patients who had previously encountered difficulties in trying to access PrEP, interviewees also recounted experiencing substantial skepticism or uncertainty about the service, especially initially. Although some of these doubts were PrEP related (eg, how would lab testing work?), others grew out of the remote or web-based modality of care and may not be specific to Nurx or PrEP. Thus, regarding telehealth generally, it is imperative to recognize that *the web-based format itself may engender barriers to be overcome*. This possibility tends to be absent from discussions of the promise of telehealth. Those wishing to use telehealth approaches (whether in health interventions or as commercial entities) should consider what information or strategies would prevent such doubts from arising in the first place, or at least how to allay them should they arise.

Another crucial point to make, based on stories interviewees shared, was that much of what influenced their assessment of Nurx lies outside of the telehealth experience itself. Especially when confronted with novel situations, users of the service drew on previous experiences to inform their notions about acceptability and appeal. For example, most users were unaccustomed to messaging back and forth in anything close to real time with a medical provider. However, such behavior was familiar from other domains (eg, text messaging and social media posts), and it seemed that not only the practice but also the meanings attached to that practice—that is, friendliness and responsiveness—became associated with Nurx. On the other hand, past *real-life* experiences were sometimes contrasted with what occurred via telehealth. In particular, most clients drew on face-to-face medical appointments as the implicit benchmark against which they evaluated their Nurx experience. As in-person appointments often came with what clients saw as hassles, such as lack of convenient appointment times, the embarrassment of talking about sexual health and practices, and providers who were not knowledgeable about or supportive of PrEP, inconveniences encountered with Nurx seemed minimal in comparison (the *trade-off*). When creating telehealth (including mobile health) services, it may be helpful to explore what previous experiences users could draw on to help them interpret their new telehealth experiences in a positive light and use design to maximize these associations of relative advantage.

In addition, although often not explicit in interviewees’ accounts, any social determinants of health perspective [27] will acknowledge that cultural and structural factors play a role in the clients’ assessment of Nurx. For instance, the degree to which clients anticipate or have experienced embarrassing or shame-inducing sexual health discussions with providers may depend on the cultural norms of the community where they live or have previously received care, which, in turn, likely undergirds how appealing it would be to avoid such encounters, and therefore how attractive clients find Nurx’s model. Examples of structural influences are geographic distribution of PrEP-knowledgeable providers, what kind of insurance clients had (if any), and whether they lived in states that had expanded Medicaid, all of which impact PrEP accessibility. To the extent that national policy influences insurance coverage (eg, both the enactment and dismantling of the Affordable Care Act), it should also be accounted for. Although it is understandable and appropriate that assessments of technology-based health interventions focus on feasibility, acceptability, process metrics, and behavioral and clinical outcomes [28-32], we urge scholars not to leave the wider social context in which telehealth is embedded entirely *outside the frame*.

One further point we wish to raise for discussion is the pressing need for fine-grained inquiry into PrEP telehealth. Our research indicated features of Nurx that were key to the appeal and users’ experience of the service, as well as revealed variation in terms of the overall balance interviewees desired between efficiency and humanity. In short, patients exhibited a range of needs and preferences for clinical encounters, and Nurx, like any technological intervention, was built to accommodate a particular range of these preferences. When these ranges overlapped, and the end goal (obtaining PrEP) was achieved, satisfaction was often the result.

A serious limitation of scholarly production on nontraditional forms of PrEP access is that it rarely engages in a meaningful way with the differences in format, service design, or context of implementation among interventions. As such, we agree with Mayer et al [7] that “studies that identify the core components of effective programmatic partnerships are needed.” They continue, however, to posit the goal of such work as the development of “normative guidance” and the promotion of “best practices for local PrEP implementation programs” [7]. We acknowledge *best practices* as potentially helpful and effective *as guidelines*, but as we have argued elsewhere [21], we believe that interventions, contexts, and users are mutually constitutive, which is to say that, in different contexts or with different users, even the same telehealth intervention might mean something different and hence function quite differently [33,34]. Indeed, *best practices* may vary so widely across different locales, key populations, or user orientations (eg, to clinical interactions) so as to preclude a singular *right* answer about *how to do* telehealth. Even within the same context, clients with different needs and preferences than the ones we interviewed could easily evaluate Nurx quite differently. For example, individuals with serious privacy concerns about the web or those who could not get over their worries that Nurx is a scam might be more reassured by and therefore more likely to opt for a service that featured real-time videoconference or telephone appointments with a medical provider. Different flavors of telehealth will appeal to different kinds of patients. Rather than assuming that we know how client-patients experience these interactions, researchers should strive to illuminate the mechanics at play, that is, we need to ask the following questions: What *is* the client’s experience? *How* is this experience produced? F*or what kind of clients* does this hold true? Digging into the specifics of the client experience in this way allows us to avoid fetishizing the modality of service delivery—after all, technology is a tool, not an end in itself.

### Limitations

As with any research, this study has important limitations, mostly to do with potential selection bias among our interviewees. Most obviously, people who lack access to the internet or are unaware of PrEP and/or Nurx could not have registered as Nurx users. In addition, potential users who felt the need for an intensely *hands-on* experience with a medical provider around PrEP would likely not have found Nurx services attractive in the first place, and those who experienced but were not able to overcome strong skepticism about Nurx probably would not have registered as users on the site. Thus, we were unable to interview anyone representing these groups.

Among the individuals we did interview, several notable categories of potential clients may be underrepresented. First, none of our interviewees identified as American Indian or Alaskan Native, Native Hawaiian and other Pacific Islander, or transgender. The latter is particularly lamentable because people of trans experience figure among those for whom telehealth has been proposed as a way to circumvent barriers to care [35]. Second, although we opted to recruit *PrEP requesters* (rather than only those who had received PrEP through Nurx) in a bid to reach individuals who had negative experiences, those users may have been less likely than their satisfied counterparts to be interested in participating in this research. However, as our analysis suggests that patients with different needs may gravitate to other ways of obtaining PrEP (via a different approach to telehealth or in a brick-and-mortar context), this simply means that other studies should pursue a similar detailed line of inquiry about patient experiences with those other methods. By comparing and contrasting our findings, we may be able to derive key characteristics or needs that would indicate how and for whom differently designed services work best. Finally, we recognize that users who lacked other ways to access PrEP may have been especially likely to report feeling positive about Nurx, even if they experienced challenges in using the service. However, this would be true of *any* PrEP service when a user has no other mode of access, and that other options are unavailable or unappealing is part of what produces satisfaction with real-world clinical settings. While acknowledging this, we do not believe it undermines our analysis, as it is focused primarily on understanding not how many interviewees said they were satisfied but how that satisfaction is produced.

### Conclusions

Interviewees recognized the web-based nature of Nurx, with its potential for real-time communication and use of automation, as a unique platform that enabled a novel form of PrEP access. However, there was variation among clients in the efficiency-humanity balance they wanted Nurx to strike. Some clients very heavily valued efficiency and seemed to see Nurx as almost analogous to other commercial entities with whom they might have a subscription (eg, Netflix). Others demanded a more personal touch and seemed to interpret the relationship they had with Nurx—technological mediation notwithstanding—as a caring one. However, for all clients, every step in the Nurx journey was simultaneously technological, clinical, and social, informed by previous experiences in various domains (eg, in-person medical appointments and texting), as well as cultural and structural considerations. While telehealth is not a panacea, having multiple, differently designed access options available may fit the needs of the broadest swath of potential users, thereby opening new spaces for therapeutic engagement.

## Abbreviations

PCP: primary care provider
PrEP: pre-exposure prophylaxis
STI: sexually transmitted infection
UCSF: University of California, San Francisco
